# Uveal Melanoma: Comprehensive Review of Its Pathophysiology, Diagnosis, Treatment, and Future Perspectives

**DOI:** 10.3390/biomedicines12081758

**Published:** 2024-08-05

**Authors:** Merve Kulbay, Emily Marcotte, Raheem Remtulla, Tsz Hin Alexander Lau, Manuel Paez-Escamilla, Kevin Y. Wu, Miguel N. Burnier

**Affiliations:** 1 Department of Ophthalmology & Visual Sciences, McGill University, Montreal, QC H4A 3S5, Canada; merve.kulbay@mail.mcgill.ca (M.K.); raheem.remtulla@mail.mcgill.ca (R.R.); tsz.h.lau@mail.mcgill.ca (T.H.A.L.); manuel.paez-escamilla@mail.mcgill.ca (M.P.-E.); 2 McGill University Ocular Pathology and Translational Research Laboratory, McGill University, Montreal, QC H4A 3J1, Canada; emily.marcotte@mail.mcgill.ca; 3 Cancer Research Program, Research Institute of the McGill University Health Centre, Montreal, QC H4A 3J1, Canada; 4 Department of Surgery, Division of Ophthalmology, University of Sherbrooke, Sherbrooke, QC J1G 2E8, Canada; yang.wu@usherbrooke.ca

**Keywords:** uveal melanoma, circulating hybrid cells, liquid biopsies, artificial intelligence

## Abstract

Uveal melanoma (UM) is the most common intraocular malignancy in adults. Recent advances highlight the role of tumor-derived extracellular vesicles (TEV) and circulating hybrid cells (CHC) in UM tumorigenesis. Bridged with liquid biopsies, a novel technology that has shown incredible performance in detecting cancer cells or products derived from tumors in bodily fluids, it can significantly impact disease management and outcome. The aim of this comprehensive literature review is to provide a summary of current knowledge and ongoing advances in posterior UM pathophysiology, diagnosis, and treatment. The first section of the manuscript discusses the complex and intricate role of TEVs and CHCs. The second part of this review delves into the epidemiology, etiology and risk factors, clinical presentation, and prognosis of UM. Third, current diagnostic methods, ensued by novel diagnostic tools for the early detection of UM, such as liquid biopsies and artificial intelligence-based technologies, are of paramount importance in this review. The fundamental principles, limits, and challenges associated with these diagnostic tools, as well as their potential as a tracker for disease progression, are discussed. Finally, a summary of current treatment modalities is provided, followed by an overview of ongoing preclinical and clinical research studies to provide further insights on potential biomolecular pathway alterations and therapeutic targets for the management of UM. This review is thus an important resource for all healthcare professionals, clinicians, and researchers working in the field of ocular oncology.

## 1. Introduction

Uveal melanoma (UM) is the most common primary intraocular malignancy in adults, with an incidence of nearly 5 cases per million individuals in the United States [1,2]. Mortality from metastatic UM was shown to be of 30% at 5 years in a Swedish cohort [3]. Furthermore, 40% fatality rates in UM were shown in patients 10 to 15 years following primary diagnosis [4,5,6]. Colorectal and liver metastasis are the leading causes of death in UM patients [3]. Furthermore, UM comes with a psychosocial burden on affected patients, with a great impact on their quality of life [7,8]. Although numerous advances in the treatment of UM have been made over the past few years, such as the development of tebentafusp—an immune system regulator—the prognosis of UM remains poor [9,10,11]. Current beliefs are in favor of the presence of micrometastatic disease at the time of diagnosis, as a result of the hematogenous spread of cancer cells. Delays in diagnosis and treatment are suggested to be major contributors to metastatic disease as well [12]. Therefore, there is an urgent need to identify novel therapeutic targets in combination with highly sensitive and specific diagnostic tools.

UM originates from the pigmented melanocytes of the uveal tissues, which consist of the iris, ciliary body, and choroid [13]. Frequent mutations in the *BAP1* (i.e., BRCA-1 associated protein 1), *EIF1AX* (i.e., eukaryotic translation initiation factor 1A X-linked), *GNA11* (i.e., guanosine nucleotide-binding protein alpha-11), *GNAQ* (i.e., guanosine nucleotide-binding protein Q), and *SF3B1* (i.e., splicing factor 3b subunit 1) genes were shown to occur in the majority of UM cases, as well as contribute on different levels to the metastatic risk [14,15]. Mutations in the GNAQ and GNA11 signaling pathways are known to drive neoplastic growth and proliferation through RAS and PI3K molecular signaling networks [16,17]. Furthermore, recent studies have underscored the importance of circulating neoplastic-immune hybrid cells (CHCs) in the blood—corresponding to dual nature hybrid cells (DNCs) in the primary tumor—and tumor-derived extracellular vesicles (TEVs) in the pathogenesis of metastatic UM [18,19,20,21,22,23]. CHCs, generated by cell fusion, are neoplastic cells that express combined neoplastic and immune cell features, which makes their identification possible through co-expression of tumor and leukocyte cell-surface markers [20,21,24]. Their presence in the bloodstream of affected patients can be used to assess metastatic UM risk. The emergence of novel diagnostic modalities, such as liquid biopsies, which consist of obtaining from the peripheral blood circulating tumor cells and other derived molecules, has opened doors to better patient care and disease outcome [25]. Herein, we will review the pathogenesis of UM, with an emphasis on novel molecular targets for diagnosis and treatment, as well as its clinical presentation, prognostic factors, and current diagnostic modalities. The contribution of liquid biopsies within the evolving field of artificial intelligence will further be discussed. Overall, this review will bridge the fundamental concepts underlying the tumorigenesis of UM with its clinical presentation and management, hence being a valuable tool for researchers and clinicians within the field.

## 2. Pathophysiology

**Figure 1:**
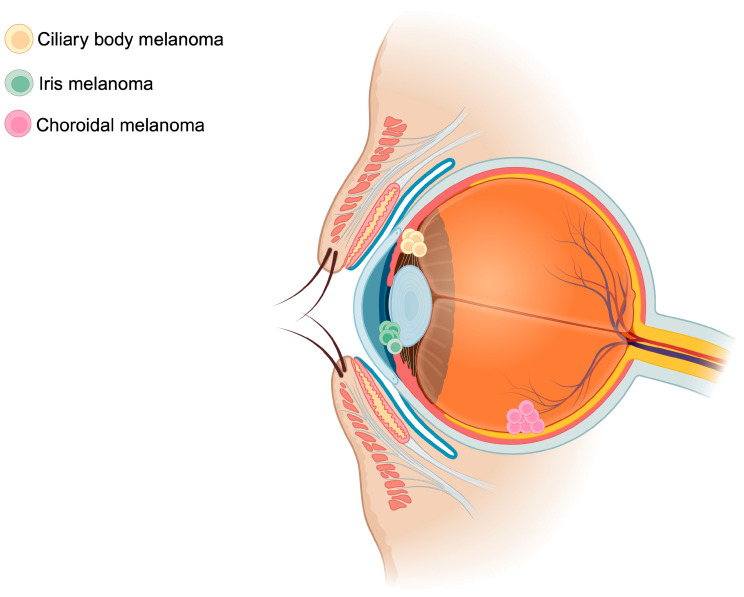
Schematic representation of uveal melanoma locations. Uveal melanomas arise mainly from the choroid (90%), followed by the ciliary body (7%), and finally from the iris (2%). The figure was created with BioRender.com.

UM arises from oncogenic mutations within the melanocytes of the iris (2% of cases), ciliary body (7% of cases), or choroid (90% of cases)—all representing distinct uveal tissues (Figure 1) [26]. The genomic instability ensuing this phenomenon promotes tumor growth and proliferation and subsequent hematogenous spread. In the ensuing section, risk factors of UM, the genetic landscape, an overview of current pathophysiology of UM, and the role of TEVs and CHCs will be discussed.

### 2.1. Risk Factors

Unlike skin melanoma, UM is not directly associated with sun exposure. Additional common risk factors for UM are the presence of an atypical cutaneous nevi, occupational exposure to irritants (e.g., cooking, welding), fair skin color, light eye color, iris nevi, nevus of Ota, periorbital dermis, and cutaneous freckles [27,28].

### 2.2. Genetic Landscape

The genetic landscape of UM has been thoroughly studied over the past years. Oncogenic mutations of *GNAQ* and *GNA11* were shown to be involved in the vast majority of UM cases [29,30]. These genes are involved in the transcription of the alpha subunit of heterotrimeric proteins (e.g., G proteins, Gq, and G11) [15]. Dysregulations in G-protein-mediated signaling pathways alter biomolecular networks involved in cell metabolism, cell proliferation, and tumor growth, which are mainly under the regulation of MAPK, PI3K, and mTOR signaling [31]. Inactivating somatic mutations of the *BAP1* gene were also shown to be involved in the majority of metastatic tumor cells and were shown to be the second leading oncogenic mutation following the *GNAQ/GNA11* genes [32,33]. BAP1—a member of the deubiquitinase superfamily of enzymes—regulates maturation and turnover of ubiquitin. Defects in BAP1 disrupt DNA replication, DNA repair, calcium homeostasis, cell proliferation and differentiation, and cell metabolism [34]. Finally, *EIF1AX* and *SF3B*, other commonly found oncogenic mutations, account for less than 25% of known cases [35,36]. A recent comprehensive literature review has thoroughly discussed the genetic and epigenetic features of UM, which we suggest as a reference for further details [37], in conjunction with additional high-quality reviews [17,38,39,40].

### 2.3. Current Knowledge on Uveal Melanoma Tumorigenesis

**Figure 2:**
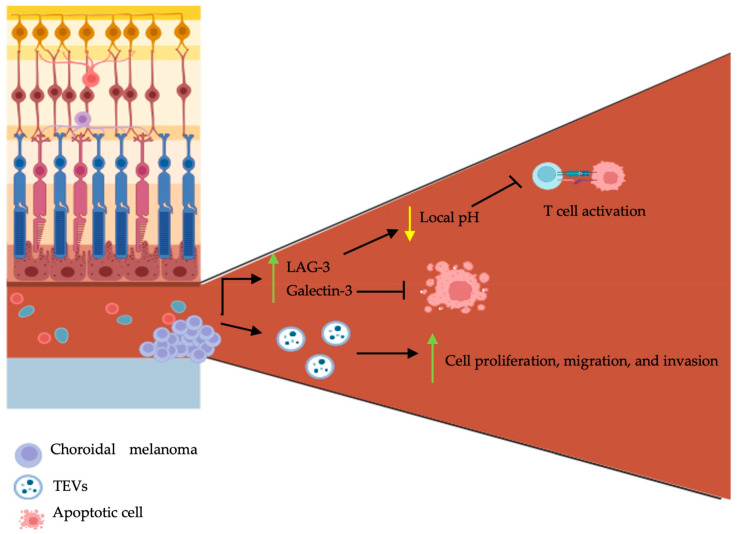
Schematic representation of choroidal melanoma tumor microenvironment, tumor-derived extracellular vesicles, and circulating hybrid cells on pathogenesis. Choroidal melanomas are characterized by central angiogenesis and peripheral tumor cell growth. They secrete LAG-3, galectin-3, and tumor-derived extracellular vesicles (TEVs). LAG-3 and galectin-3 inhibit T cell activation and cancer cell apoptosis respectively. TEVs promote choroidal melanoma proliferation, migration, and invasion. The figure was created with BioRender.com.

The majority (90%) of UMs arise from the choroid of the uveal tract [41]. The eye is in fact an immune privileged site; ocular immune privilege is defined by the presence of local and systemic mechanisms to limit local inflammation [42]. Dysregulations of the immune system are directly associated with the development of malignancies. In contrast to other solid tumors, where acute inflammation is known to induce cancer cell death [43], the inflammatory cascade in UM favors tumorigenesis. The inflammatory phenotype of UM, which is defined as a lymphocytic inflammatory process, is known to be associated with a poor prognosis [44,45,46,47,48]. Lymphocytic infiltration is marked by an increase in lymphocytes, macrophages, and HLA class I and II expression. The nuclear factor-κB (NFκB) is a known regulator of the innate and adaptative immune system, with a significant role in inflammation homeostasis [49]. Activation of the NFκB pathway was found to occur in primary and metastatic UM, subsequently upregulating UM cell proliferation and inhibiting apoptosis [50]. Furthermore, it was shown that the metastatic potential of UM cells was acquired through transendothelial migration [50]. It was recently demonstrated that the oncogenic mutations activating Gαq/11 induced the activation of the guanine nucleotide exchange factor, TRIO [51]. Numerous cytokines, chemokines, and the role of tumor-associated macrophages were previously thoroughly covered by Amaro et al. [52], which will not be covered again within this manuscript. However, the role of the microenvironment, TEVs, and CHCs—novel concepts in the pathogenesis of UM—are discussed within the ensuing section (Figure 2).

### 2.4. The Role of Tumor-Derived Extracellular Vesicles and Circulating Hybrid Cells: Novel Advances

The tumor microenvironment (TME) possesses complex roles and is mainly involved in cell communication and tumoral support. It encompasses various cellular and non-cellular components, such as soluble mediators (e.g., cytokines, chemokines, and growth factors), various stromal cells (e.g., endothelial cells, fibroblasts, and immune cells), and biomolecular markers [53]. There exists a connected interplay between the tumoral cells and the TME; as cancer cells influence the composition of the TME, the latter further influences the growth, progression, and metastasis of cancer cells [54]. A recent study aiming to better characterize the TME of UM has shown that tumor cells grow with a specific configuration: angiogenesis occurs in the central part of the tumor, whereas immune cells are disturbed along the outer parts [55]. Secondly, a high expression of lymphocyte activating gene-3 (LAG-3) and galectin-3 was found in the enucleated eyes of patients with UM following immunohistochemistry [55]. LAG-3 is an immune checkpoint regulator shown to be highly expressed on exhausted T cells in the TME’s [56]. It is involved in inhibitory signal transmission to T cells, which subsequently renders these cells non-functional [56]. It was shown that LAG-3 binds the T cell receptor (TCR)-CD3 complex on CD4^+^ and CD8^+^ T cells and further inhibits the interaction of the TCR-CD3 complex with Lck, a member of the Src kinase family, by lowering the local pH [57]. Similarly, galectin-3 is also involved in immunosuppression within the TME [58]. In human bladder carcinoma cells, overexpression of galectin-3 was shown to inhibit TRAIL-induced apoptosis through the upregulation in constitutive expression of Akt and subsequent inhibition of BID cleavage [59].

A major constituent of the TME are TEVs, which are known to bridge the communication between tumor cells and their microenvironment. TEVs favor tumorigenesis through the transfer of their material to neighboring cells [60]. They are cell-secreted vesicles that encompass various bioactive substances, such as nucleic acids (e.g., DNA and RNA), lipids, proteins, and metabolites [61]. Studies have shown that the concentration of UM-derived EVs in the blood is comparable to that found in the aqueous humor and vitreous humor [62]. Therefore, given their abundance in peripheral blood and subsequent ease in obtaining samples for testing, they have thus become an exciting avenue as a diagnostic tool. The oncogenic potential of TEVs in metastatic disease has been proposed as a mechanism for UM dissemination. Tsering et al. have shown that in vitro exposure of Fibro-BKO (i.e., *BRCA1*-deficient fibroblasts) cells to UM-derived EVs significantly increased tumor cell proliferation, migration, and invasion [23]. Similar results were obtained in an in vivo model: subcutaneous injection of Fibro-BKO cells exposed to UM-derived EVs in SOD/SCID mice induced tumor growth [23]. Analysis of TEV components demonstrated that UM-derived EVs encompassed proteins involved in cell–cell adhesion, leukocyte transendothelial migration, cell division and migration, cell signaling pathways (e.g., MAPK, vascular endothelial growth factor receptor (VEGFR), and Wnt), and in metastatic niche formation (e.g., integrin αV, GNAQ, and GNA11). In vitro UM-derived EV uptake in hepatocytes was further shown to enhance the phosphorylation of MET, ERK, AKT, and STAT3 in a dose-dependent manner [63]. The expressions of chaperone molecules (HSPB1), alpha-enolase (ENO1), and chemo-attractants were also upregulated [23]. UM-derived EVs were shown to upregulate the expression of interleukin (IL)-β, IL-8, and growth factors (i.e., fibroblast growth factor 1 (FGF1), VEGF, and tumor growth factor (TGF) β) [63]. Similarly, UM-derived EVs were shown to induce hepatic remodeling through hepatic stellate cell activation—demonstrated by an increase in their proliferation and metabolism—increased capillary-like networks in endothelial cells, and hepatic fibrosis, which are considered hallmarks in hepatic pre-metastatic niche formation [64,65]. Fibronectin 1 (FN1), a widely known marker involved in pre-metastatic niche [66], was shown to be upregulated in hepatocytes with UM-derived EV uptake [63].

CHCs are a novel tumor cell population found within the TME recently identified in the blood of patients with UM [20]. They highly express the markers gp100 (a melanocytic marker), HTR2b (a cell surface serotonin receptor), and CD45 (a leukocyte common antigen) [18]. They result from the fusion of tumor cells with leucocytes and form the backbone of tumor heterogeneity [21,67,68,69,70,71]. It was shown in other cancers that the fusion of tumor cells with macrophages induces a hybrid that exhibits a mixed phenotype and differential response to the TME [21]. To demonstrate the role of CHCs in tumor progression, Dietz and colleagues harvested CHCs from a murine mammary tumor model, dissociated the cells in vitro, and subsequently injected the single-cell suspension into the mammary fat pad [20]. It was shown that the single-cell suspension generated from CHCs supported neoplastic growth [20]. CHCs could be used as a novel tool to stratify prognostic risk, as they were shown to be present in the peripheral blood of all patients with stage 1 to stage 3 UMs [18]. Their relatively high prevalence, cost-effective quantification, and less invasive sampling through liquid biopsies provide an exciting venue for research.

## 3. Epidemiology of Uveal Melanoma

### 3.1. Classification Systems

There are numerous classification schemes for UM, with the most used being the American Joint Committee on Cancer (AJCC) TNM system and the clinical classification of the collaborative ocular melanoma study (COMS) [72,73,74]. However, additional classification methods include systems based on genetic testing (i.e., The Cancer Genome Atlas (TCGA) classification) [75,76], histopathological features (i.e., modified Callender’s classification system) [77,78], and gene expression profile classification [79].

The AJCC TNM system for UM staging further encompasses prognostication [80,81]. The extent of the primary tumor (T) is determined based on the tumor’s largest basal diameter, thickness, the presence of ciliary body involvement, and extraocular extension. The N and M staging refers to regional lymph node involvement and the presence or not of distant metastasis, with subsequent metastatic tumor dimension, respectively [80,81]. However, it is to be noted that UM dissemination mainly occurs through the hematogenous route, given the absence of lymphatics within the eye. Extension to surrounding structures, such as the conjunctiva, can allow for lymphatic dissemination [82].

### 3.2. Clinical Presentation

The clinical presentation of UM varies according to tumor size and location. In many cases, UM detection and diagnosis is an incidental finding during routine ocular exams, given that most patients are asymptomatic at presentation [83,84]. However, in the presence of visual symptoms, UM can be missed by clinicians in up to 23% of cases [83]. The most common visual symptoms reported by patients include blurry vision, photopsia, visual field defects, floaters, and rarely, eye pain and metamorphopsia [84,85].

### 3.3. Prognostic Factors

Survival rate for UM depends on a variety of factors, ranging from clinical, molecular, histopathological, and genetic characteristics (Table 1). Tumor location influences prognosis, with tumors located within 1 mm of the optic nerve head (juxtapapillary) or having any tumor portion located in the ciliary body being associated with a low survival rate [86,87]. Tumor location is also known to influence time of disease detection; iris melanomas are typically diagnosed one to two decades earlier, which may contribute to lower metastasis rates and greater prognosis [88,89,90]. Another important feature to consider during prognostication is somatic mutations. Risk of UM metastasis has been shown to be linked to few somatic mutations in the presence of chromosome 3 monosomy or partial monosomy [91]. Increased risk of metastasis was shown in tumors harboring 6p loss, 6q loss, 8p loss, and 8q gain, whereas the risk of metastasis was decreased in tumors with 6p gains [91].

Donald Gass was the first to identify certain clinical and multimodal imaging characteristics of indeterminate nevi that could pose a risk to grow and undergo malignant transformation [92]. Further studies delved into these characteristics in their landmark study of indeterminate nevi [74,93]. More recently, Shields et al. have suggested a mnemonic (i.e., To Find Small Ocular Melanoma-Using Helpful Hints Daily (TFSOM-UHHD)) to help differentiate choroidal nevus from UM (Table 2) [94]. The presence of 3 or more factors is most likely associated with UM.

## 4. Diagnosis

### 4.1. Ocular Findings

**Figure 3:**
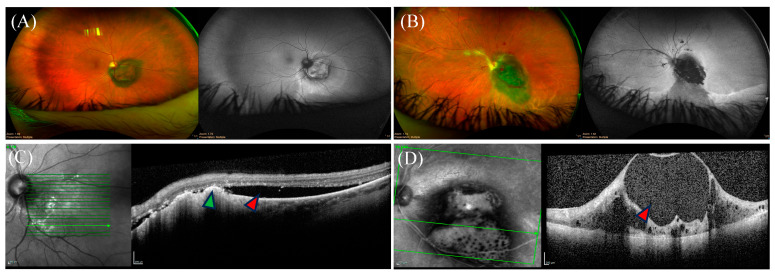
Ocular findings of posterior uveal melanoma on imaging. (**A**,**B**) Coloured fundus photos of two distinct juxtapapillary uveal melanoma (UM) with their corresponding monochromatic photography on the right of each panel. Representative optical coherence tomography (OCT) images are shown below the corresponding panels. (**C**) OCT imaging showing subretinal fluid (red arrow) with drusen deposits (green arrow). (**D**) OCT imaging depicting disorganized inner retinal layers in a cystoid configuration (red arrow).

Posterior UMs typically present as an elevated choroidal mass on fundus examination, with a varied degree of subretinal fluid and orange pigment (Figure 3). When the tumor invades and penetrates Bruch’s membrane, the typical mushroom-shaped appearance can be observed [95].

### 4.2. Optical Coherence Tomography

Optical coherence tomography (OCT) is a non-invasive, radiation-free imaging modality that provides high spatial resolution and allows detailed imaging of the retina by creating a cross-sectional map of the retinal layers [96]. On OCT, posterior UM can be associated with photoreceptor or ellipsoid zone (EZ) loss, RPE loss or hyperplasia, intraretinal or subretinal fluid, retinoschisis, retinal thinning, outer plexiform layer (OPL) splitting, bacillary layer detachment, or subretinal hyperreflective material [97].

### 4.3. Ultrasonography

**Figure 4:**
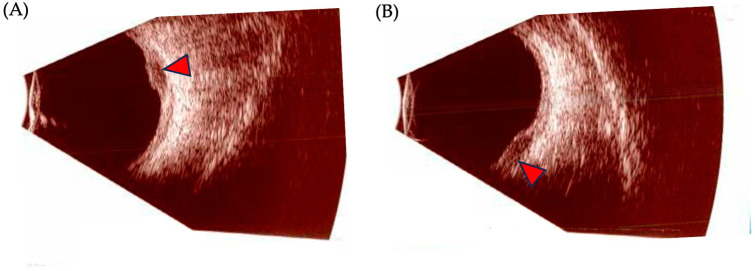
Ocular ultrasonography findings in posterior uveal melanoma. (**A**,**B**): B-scan images of a posterior uveal melanoma (UM). Red arrows depict the tumoral mass.

Ocular ultrasonography is the most useful ancillary test to diagnose, differentiate, and follow the progression of UM (Figure 4). Its two modalities, A-scan (amplitude) and B-scan (brightness), offer useful information and allow for the measurement of internal reflectivity, largest basal diameter (LBD), and apical height/thickness. On a standardized A-scan, the tumor shows low-to-medium internal reflectivity in a decrescendo fashion, with internal vascularity seen as fast movement of internal spikes. On a B-scan, the tumor is usually dome shaped, or if it has broken through Bruch’s membrane, it appears as a mushroom-shaped tumor with an adjacent localized exudative retinal detachment. Other features include acoustic hollowness and choroidal excavation [98].

### 4.4. Magnetic Resonance Imaging

Magnetic resonance imaging (MRI) is a non-invasive technology that generates tridimensional images. MRI can be a useful tool for the diagnosis and monitoring of choroidal UM [99]. Key features of choroidal UM that can be detected on MRI include evidence of a nodular-like lesion, which can be associated with retinal detachment [100]. Posterior UM appears hyperintense on T1-weighted images and hypointense on T2-weighted images, with additional diffusion restriction features [100].

### 4.5. Histopathology

**Figure 5:**
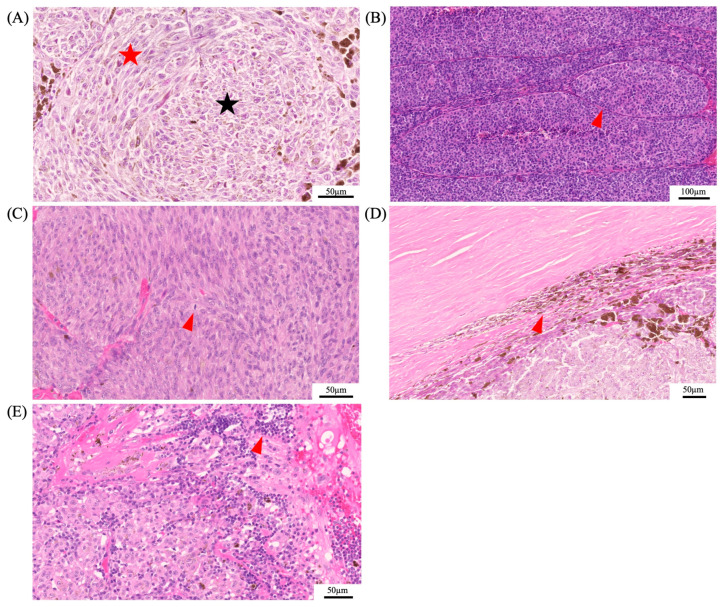
Histopathological features of uveal melanoma. Panel (**A**) depicts the histopathological differences of spindle cells (red star) and epithelioid cells (black star). Epithelioid cells are associated with a poor prognosis. Additional histopathological features that were shown to be associated with a poor prognosis are the presence of vascular loops (red arrowhead) (**B**), mitotic figures (red arrowhead) (**C**), extraocular involvement (red arrowhead) (**D**), and lymphocytic infiltration (red arrowhead) (**E**).

In 1931, Callender established the histopathological classification for UM, dividing it into five categories according to cell type: spindle A, spindle B, mixed, fascicular, and epithelioid [101]. The modified Callender classification grouped these categories into three, consisting of spindle-cell melanoma (>90% spindle cells), mixed-cell melanoma (mixture of spindle and epithelioid cells), and epithelioid-cell melanoma (>90% epithelioid cells) [77]. Cell type has one of the highest associations with survival, along with the genetic profile. Spindle-cell melanomas have the greatest chance of survival past 5 years, whereas mostly epithelioid-cell melanomas have a low survival rate and are characterized by a greater-sized nuclei and an absence of overall cohesiveness [77,102]. Other histopathological features that were shown to be associated with poor outcome are size, the presence of vascular loops, mitotic figures, extraocular involvement, and lymphocytic infiltration (Figure 5) [103,104].

## 5. Liquid Biopsies as a Novel Diagnostic Tool

Conventional methods of biopsies for UM, such as fine needle aspiration biopsies, have posed significant challenges. Given the intraocular and subretinal localization of the tumor, there is an inherent risk of rhegmatogenous retinal detachments and persistent vitreous hemorrhage [105]. Furthermore, direct biopsy of the tumor may result in extra-ocular extension or seeding through the needle [106]. As with all operations in the eye tissue, biopsies of UMs are also associated with endophthalmitis and have even been shown to be associated with worsening visual acuity in 13% of patients [107,108,109,110]. Furthermore, the prognostic yield of these biopsies is often limited, especially if the tumor is small or posteriorly located [109].

Given the inherent risks and low efficacy of standard biopsies, significant interest was dedicated to liquid biopsies. The majority of the focus in the field of liquid biopsies has been towards blood and serum-based analysis; however, urine, cerebrospinal fluid, ascites, saliva, and even aqueous and vitreous humor have also garnered attention [25]. Liquid-based biopsies are simple to conduct and pose a lower risk than conventional biopsy methods of the primary tumor [111]. Furthermore, biopsies of the primary tumor may fail to recognize secondary or metastatic disease or capture the heterogeneous nature of the tumor [25]. Tumor markers identified in liquid biopsies include CTCs, DNA, RNA, and proteins [25,111] (Table 3).

CTCs have been identified in multiple malignant conditions such as breast cancer, prostate cancer, and UM, which have provided clinicians with pertinent prognostic data on disease metastasis [112,113]. It is believed that these CTCs may represent the seeding of tumors before metastatic disease [125], as described in previous sections. Since CTCs in UM patients are rare, isolation methods are required [111]. The most common way to isolate and capture CTCs is through immunomagnetic and filtration-based enrichment. Immunomagnetic enrichment takes advantage of tumor protein expression, whereas filtration-based enrichment relies on size and compressibility differences between CTCs and blood cells [126,127]. There is a statistically significant difference in UM basal diameter, tumor height, progression-free survival, and overall survival between patients with more than 10 CTCs/10 mL of blood compared to those with lower CTC concentrations in non-metastatic patients [114]. Immunomagnetic isolation has become the preferred methodology of isolating CTCs with multiple different antibody-bound magnetic particles available on the market [111]. Although variations exist among the different antibodies available, the one consistency is that increasing CTC counts are associated with worsening prognosis [111]. There is also evidence that suggests that copy number variants in the primary tumor and CTCs have similar chromosomal aberrations (i.e., somatic chromosomal copy number alterations (SCNAs)) [115]. Comparative analysis of SCNAs in CTCs isolated from the peripheral blood of a patient with metastatic UM or in peripheral blood mononuclear cells (PBMCs) demonstrated the importance of tumor-associated chromosomal aberrations; primary UM tumors and CTCs had showcased various chromosomal gains and losses, whereas PBMC lacked SNACs [115]. Therefore, evaluation of these chromosomal aberrations in primary tumors and CTCs can significantly contribute to disease prognostication. Importantly, distinguishing between UM and nevi has been a challenging task, and as demonstrated by Bande et al., 50% of patients with UM had more than 1 CTC compared to none in patients with nevi [121]. Mazzini et al. found that more than half of patients with UM have CTCs, whereas patients with choroidal nevi had none [114]. Callejo et al. found that in 30 patients diagnosed with UM, all had detectable CTCs [113]. However, it should be noted that the half-life of CTCs is low, with it being between 1 and 2.5 h [128,129]. With improvements in CTC isolation and standard genomic techniques such as shallow whole genome sequencing (sWGS) and fluorescence in situ hybridization (FISH), it is likely that CTC genomics will play a role in prognosticating UMs [115,116]. It should be noted that the prevalence of CTCs in UM patients in the literature may vary depending on the methodology by which CTCs are isolated. These variations can include, but are not limited to, sampling from arterial or venous blood and the antibodies employed [25].

Circulating tumor DNA (ctDNA) is DNA that has either been actively secreted from a tumor cell or released through cell necrosis or apoptosis [111,130]. Pathological markers in the primary tumor have been identified in the ctDNA of patients with prostate cancer, as well as other malignancies [111,131]. Similar findings have been demonstrated in UM, where ctDNA monitoring predicted disease response and metastatic UM progression, and the use of next generation sequencing of ctDNA helped to predict the response to protein kinase inhibitor therapy [117]. In addition, ctDNA in UM has been used to predict treatment response to checkpoint inhibitors [118] and in animal models, ctDNA from UM preceded clinical detection of the disease [132]. Bidard et al. demonstrated that 22 out of 26 patients with UM had ctDNA and that higher ctDNA levels were correlated with hepatic miliary metastases and tumor volume [133]. Interestingly, it was also demonstrated that ctDNA was correlated with CTCs and progression-free survival [133]. It should be noted that isolating ctDNA can be challenging as circulating free DNA from physiologic processes is more abundant [25]. GNAQ and GNA11 have been evaluated in detail as they can be used to differentiate ctDNA from circulating free DNA [25,134]. Methodologies for isolating ctDNA will likely improve their clinical yield in liquid biopsies.

Other than circulating DNA, circulating micro-RNA (miRNA) has also been shown to have clinical potential in managing UM. Circulating miRNA was used initially in breast cancer, as isolating these CTCs was challenging and certain miRNA were found to be upregulated [135]. The clinical advantage of miRNA is its long half-life, ease of accessibility, as well as high specificity and sensitivity [25,111]. Increased plasma miRNA-618 and decreased vitreous miRNA were identified in patients with UM when compared to healthy controls [119]. It is also noted that miRNA regulation differs in patients with metastatic vs non-metastatic disease [120].

Blood-based proteomic data have demonstrated potential clinical value in the prognostication of UM. Melanoma-specific gp100 and cathepsin have been elevated in patients with UM in comparison to healthy controls [121,122]. Proteins involved in extracellular matrix remodeling as well as cancer migration and invasion have been shown to be upregulated in patients with high risk for UM [121]. Both heat shock protein 27 and osteopontin assays can differentiate between metastatic and non-metastatic UM post-treatment [123]. Multiplexed analysis of protein abundance of a multitude of suspected proteins demonstrated an area under the curve (AUC) of 91% in the detection of metastatic disease [136]. Analysis of hepatic biomarkers has also been suggested to identify metastatic incidence to the liver, although this has limited clinical value in early prediction of metastatic potential [111]. Furthermore, it has been hypothesized that TEVs can also be employed to evaluate UM. However, there is limited clinical evidence of their effectiveness currently, with most studies representing small trials or being performed on non-human samples.

Blood is not the only fluid that has been evaluated for liquid biopsy in UM. Aqueous and vitreous samples have also been studied [119,137,138]. These methods have a higher inherent risk and are more invasive. It is likely for this reason that they have not been the subject of extensive research. However, Im et al. demonstrated that SCNAs were identified in patients with UM from aqueous humor samples and showed high concordance with copy number variations within the matched tumor [139]. Elevated levels of miRNA have also been identified in the vitreous of patients with UM [119]. VEGF has been shown to be elevated in the aqueous humor of affected patients with positive correlations between tumor height and diameter [137,138], and S-100 protein has been shown to be elevated in both the vitreous and aqueous humor of patients with UM [124].

Liquid biopsies may provide a non-invasive, easily available, quantifiable metric for monitoring and prognosticating patients with UM. It is likely that further studies in this field will provide available clinical tools to guide patient care. However, limitations exist, making their near-future clinical application difficult. In early clinical presentation, the sensitivity for ctDNA detection is poor given low concentrations in peripheral blood. Furthermore, the isolation of TEVs requires an analysis platform, such as ELISA, fluorescence-activated cell sorting (FACS), or nanoparticle tracking analysis (NTA) [140]. However, FACS analysis can be costly and was shown to exhibit inconsistency in exosome detection [141], whereas NTA requires a significant processing time, making its translation to clinical practice much more difficult [142].

## 6. Bridging Artificial Intelligence with Diagnostic Tools for Uveal Melanoma

In the management of malignant diseases, diagnosis and prognostication has always been the primary step. However, this has been a challenge for certain malignant diseases, particularly in the eye. Fortunately, advancements in computer power and novel approaches to machine learning have allowed for the development of clinical decision-making tools based on large data collections.

As the most common primary intraocular malignancy of the eye in adults with multiple available imaging modalities, UM has been an interest of study for machine learning applications. Of particular interest, differentiating between a benign choroidal nevus and malignant choroidal melanoma has been a clinically difficult task, for which machine learning applications have been applied [143]. In 2022, Zabor et al. evaluated 123 patients with small choroidal melanocytic tumors and trained a model using lasso logistic regression to predict malignant growth [144]. Clinical features included gender, tumor height, subretinal fluid, orange pigmentation, and distance from the optic nerve [144]. The initial model demonstrated an AUC of 0.880, and when tested against an external data set of 240 patients, the AUC was 0.861, demonstrating minimal overfitting [144].

Histopathological findings in UM have been used to prognosticate survival and metastatic potential. By leveraging machine learning applications in histopathology, attempts have been made to improve these predictions [143]. In one pilot study of 20 patients, the genetic expression profile of a tumor was identified from the digital cytopathology images with a 75% accuracy [145]. In a follow-up study employing dual-attention feature extraction of 82 patients, the presented model had a 91.7% accuracy, sensitivity, and specificity for predicting the genetic expression profile from cytopathology images [146].

Beyond diagnosis, machine learning applications have been applied for predicting metastatic potential and long-term survival [143]. In 2022, Chen et al. leveraged random forest to develop two classifier models [147]. The first model was aimed at predicting long-term survival in UM patients, demonstrating an AUC of 0.882, and the second predicted metastasis with an AUC of 0.846 [147]. Conversely, Luo et al. also employed random forest models to predict survival and risk of metastasis in UM patients who had undergone plaque brachytherapy, primarily using clinical measures [148]. This model had an AUC of 0.880, with a total accuracy of 83% for predicting survival and an AUC of 0.850 with a total accuracy of 79.5% in metastasis [148]. Studies that incorporate both histopathological findings and clinical findings appear to have even more promising results. Damato et al. developed a neural network to predict time to patient death based on coronal tumor location, sagittal tumor location, anterior tumor margin, largest basal tumor diameter, and cell type in 2543 patients [149]. The network error in predicting death was 3.8 years compared to the error of 4.3 years in the clinician group [149]. Using back propagation, Kaiserman et al. were able to develop a neural network capable of predicting five-year survival rates of UM patients treated with brachytherapy with an 84% accuracy [150]. Using histopathological images alone, deep learning methodologies have been applied to predict the prognosis of patients with over 90% accuracy for subsets of data [151]. It should be noted that attempts have been made to incorporate data from liquid biopsies. Song et al. used logistic regression to analyze serum biomarkers for the diagnosis of UM and for the prediction of metastasis [123]. They identified that heat shock protein 27 and osteopontin in a two-maker panel had an AUC of 0.98 in diagnosing UM and an AUC of 0.78 in predicting metastatic disease [123].

Although some evaluated features were associated with the target outcomes, there has been limited research into feature importance [147]. Feature importance has allowed computer scientists to evaluate which features are most important to a neural network in predicting the target outcome [152]. This information may help guide physicians in identifying new features for prognosticating UM. In addition, most neural networks have relied on clinical or histopathological data. Few studies have evaluated fundus photography or radiographic imaging, whereas most computer vision studies have focused on histopathological images [143,153]. Incorporating clinical imaging into machine learning studies will likely improve the functionality of these networks when incorporated with histopathological data. Finally, it should be noted that there have been very few deep learning trials, which will likely have improved functionality over the employed classical machine learning and regression models [143,151,153]. The majority of studies have small sample sizes with a few exceptions [143,149,153]. This is likely due to the rare nature of this disease. Despite this, it is still promising, as the small sample sizes have shown relative success in diagnosing the disease and predicting clinically significant outcomes. Large multicentered datasets will hopefully provide more promising results in producing clinical decision-making tools.

## 7. Current Treatment Methods

The current therapeutic landscape for UM primarily revolves around surgical interventions (i.e., tumor resection, enucleation, or exenteration), radiation therapy, photocoagulation, and transpupillary thermotherapy (TTT) (Table 4) [154].

Surgical resection involves the complete or partial removal of the tumor based on its size and location, with either the transscleral resection or the exo-resection approach [155]. This method is usually reserved for smaller tumors in the ciliary body or iris. Enucleation (i.e., removal of the globe) and exenteration (i.e., removal of the globe and its surrounding structures, such as the muscles, fat, nerves, and eyelid) are surgical approaches that are indicated in cases that present with large tumor size or extraocular involvement [156,157,158]. However, few studies have reported malignancy recurrence in cases treated with both approaches, which was subsequently associated with poor prognosis [157,159,160]. Radiation therapy entails both plaque brachytherapy and proton beam radiotherapy, which is a preferred alternative to surgical resection due to their precision in targeting the tumor while sparing surrounding tissue [161,162]. However, tumor thickness is a limiting factor for radiation therapy, given the increased risk of complications in tumors thicker than 6 mm [162]. Photocoagulation is a laser treatment technique used broadly in ophthalmology and is employed primarily for smaller tumors. Finally, TTT uses a 3 mm diode laser beam with infrared radiation to heat and destroy tumor cells through the pupils.

**Table 4:** Overview of current therapeutic approaches for uveal melanoma.

Therapeutic Approach	Method	Indication	Disadvantages	References
Surgical resection	Complete or partial removal depending on tumor size and location	Localized tumors		[155]
Enucleation	Complete removal of the globe	Thickness > 12 mm Basal diameter >18 mm Tumor seeding into the trabecular meshwork Extraocular involvement Melanoma-related glaucoma	Poor prognosis in case of tumor recurrence	[156,159,160]
Exenteration	Removal of the globe and its surrounding structures, such as the muscles, fat, nerves, and eyelid	Thickness > 12 mm Basal diameter >18 mm Multifocal or recurrent disease Painful eye Extraocular involvement	Poor prognosis in case of tumor recurrence	[157,158]
Radiation therapy	Plaque brachytherapy: Localized application of internal radiation through plaque (containing radioctive source) suturing on episclera.	Small and medium sized tumors	Associated with radiation-induced complications, such as: - Poor visual outcome - Radiation-induced cataracts - Vitreous hemorrhage - Neovascular glaucoma - Secondary glaucoma - Retinal detachment - Radiation retinopathy, maculopathy - Optic neuropathy Contraindicated in tumors < 2 mm from the optic disc and large tumors Limited adaptability of the applicator to the area, leading to increased radiation exposure	[161,162]
	Proton beam radiotherapy: Tantalum marker (beam) placement within the tumor for direct radiation for 4 days	Tumor height > 5 mm Narrow base Tumors close to optic nerve Ciliary body involvement greater than one clock hour Extraocular involvement Iris and ciliary body melanomas	Associated with radiation-induced complications (as mentioned above) Limited availability	[161,162,163,164,165,166,167]
Photocoagulation	Laser used to burn and destroy tumor cells	Small, peripheral tumors	Increased risk of recurrence Increased risk of extension through Buch’s membrane Associated with increased risk for: - Choroidal neovascularization - Macular edema - Retinal detachment - Vitreous hemorrhage	[154,168]
Transpupillary thermotherapy	Near-infrared diode laser	Small, accessible tumors	Associated with risks of: - Retinal vascular occlusions - Macular edema - Vitreous hemorrhage - Retinal detachment - Optic disc atrophy	[154,169]

## 8. Novel Therapeutic Approaches

The therapeutic treatment of UM has been a complex challenge in oncology, with limited treatment options and the possibility for UM to progress to metastasis. However, there has been significant progress over the last few years in both preclinical and clinical research, offering promising ways to improve patient outcomes.

### 8.1. Immunotherapy

Immunotherapy is a promising approach for UM treatment, which makes use of the body’s immune system to target cancer cells. Recent preclinical studies have focused on understanding the immunogenicity of UM and developing immunotherapeutic strategies to overcome immune evasion mechanisms (Table 5).

#### 8.1.1. Checkpoint Inhibitors

Checkpoint inhibitors function by acting on specific immune checkpoints. Programmed death-ligand 1 (PD-L1) is expressed in UM cells and interacts with PD-1 receptors on T cells, which subsequently inactivates T cell function, allowing tumor cells to escape the immune response. Another checkpoint protein is CTLA-4. It functions by downregulating the immune response by competing with CD80 and CD86 to bind to the stimulating receptor on antigen-presenting cells, thereby inhibiting T cell activation at the initial stage [194].

Ipilimumab (an anti-CTLA-4 monoclonal inhibitor) has been investigated at various dosages across prospective studies and retrospective analysis [195]. At 3 mg/kg body weight, the overall response rates (ORR), defined as the ratio of patients treated with the drug of interest who observed a change in tumor size on the patients who did not exhibit a change in tumor size following drug administration, was shown to range from 0 to 4.8% [170,171,172,173]. Higher doses of ipilimumab (i.e., 10 mg/kg) have been explored and showed higher prolonged median overall survival, but similar ORR compared to lower dosages [173,174,175]. However, higher doses of ipilimumab were shown to be associated with an increased risk of immune-induced colitis [196].

PD-1 inhibitors (pembrolizumab, nivolumab) have been investigated within clinical trials [176,177,178,179,180,181,182,183]. However, they have shown relative lower effectiveness as compared to when utilized in cutaneous melanoma. This may be attributed to the lower mutational burden in UM, which translates to a lower number of neoantigens recognizable by the immune system. The efficacy of pembrolizumab, ipilimumab, and a combined therapy with ipilimumab and nivolumab was compared in a retrospective population-based study [179]. None of the 24 patients who received ipilimumab responded to the therapy with a median progression free survival of 3.0 months [179]. Conversely, the PUMMA meta-analysis showed systemic therapy had a median OS of 9.3 months [197]. Another study combining nivolumab and ipilimumab had a median OS of 12.7 months [198]. The objective response rate of nivolumab and pembrolizumab varied widely among different trials. Thus, it is difficult to draw conclusions with regards to the efficacy of PD-1 blocking antibodies [199], but overall results demonstrate a better safety profile and median OS compared to CTLA-4 inhibitors. Overall, immune check-point inhibitors are not as effective for UM as compared to cutaneous melanoma, as evidenced by a poor long-term prognosis with isolated hepatic metastasis in metastatic uveal melanoma [200]. Tebentafusp is one of the most recent innovations for metastatic UM, which is an immune-mobilizing monoclonal T cell receptor (TCR) against cancer (ImmTAC; immune-mobilizing monoclonal TCRs against cancer) targeting HLA-A*0201.gp100280-288 and CD3 [10]. It is a first-in-class ImmTAC that has demonstrated greater overall survival benefits compared to other immune therapy treatments [10]. However, most patients with metastatic UM do not qualify as they are HLA-A*0201 negative.

A recent systematic review of checkpoint inhibitors on metastatic UM suggested that dual immune check-point inhibitors are more effective than single-agent therapies and could be a potential for future immune therapies [184]. Further research is investigating the combination of immune therapy with epigenetics, immune therapy with oncolytic viruses, and immune therapy with targeted genetic therapy [201]. Current trials investigating immune therapy combined with targeted therapy include pembrolizumab with olaparib (i.e., a PARP (poly (ADP-ribose) polymerase) inhibitor), pembrolizumab with LNS8801 (i.e., a G protein-coupled estrogen receptor (GPER) agonist), atezolizumab with IN10018 (i.e., a PD-L1 inhibitor with an FAK (focal adhesion kinase) inhibitor), and pembrolizumab with APG115 (i.e., an MDM2 inhibitor). Although combined immune checkpoint inhibitors demonstrate greater efficacy, it may prompt a higher number of adverse events. Thus, further research involving combinations of immune therapy will require a personalized approach.

#### 8.1.2. Oncolytic Viruses

Oncolytic viruses are engineered viruses, which specifically infect and kill cancer cells. These viruses can directly destroy tumor cells by inducing tumor lysis via a systemic immune response. An example is T-VEC (i.e., talimogene laherparepvec), a modified herpes simplex virus and FDA-approved oncolytic virus for cutaneous melanoma, which is modified to express GM-CSF and therefore increases dendritic cell function, which subsequently leads to a more effective T cell-mediated response against cancer cells [202]. Early clinical trials with T-VEC are investigating its use in treating metastatic UM for potential local control, as well as inducing a durable systemic response [203]. Current research is exploring how these oncogenic viruses can alter the tumor microenvironment to make it more susceptible to immune attack [204]. In addition to T-VED, other oncolytic viruses, such as coxsackieviruses and HF-10, are being explored for potential use in cutaneous melanoma and could potentially be applied to UM [186,187]. Recently, the successful cytotoxic effects of the oncolytic ECHO-7 virus strain were demonstrated in UM cell lines (i.e., MP41, 92-1, and Mel-202 cell lines) [185].

Oncolytic virus HSV-EGFP was shown to exhibit sensitivity and cytotoxicity towards the 92-1, MUM2B, and MP41 UM cell lines, and additional results suggested that the oncolytic HSV-1 is effective in treating UM in vitro and in vivo [188]. Ongoing investigations of other oncolytic viruses include a phase I clinical trial at Mayo Clinic, which is exploring vesicular stomatitis virus (VSV) vectors expressing interferon (IFN) β and tyrosinase-related protein 1 (TYRP1) in previously treated patients with metastatic UM via intratumoral and intravenous administration [189]. The study demonstrated clinical safety of VSV-IFNβ-TYRP1 in the target population and a dose-response immunogenicity to TYRP1 [189]. A significant area of interest is the combination of oncolytic viruses with checkpoint inhibitors [190]. This aims to enhance the overall immune response against UM by providing both the initial activation of the immune system through viral infection and the sustained activation by checkpoint blockade. Despite the limited number of current clinical trials, oncolytic viruses represent a novel therapeutic strategy for patients with metastatic UM.

More recently, virus-like drug conjugates (VDCs) have demonstrated great potential for selective delivery of drugs to tumor cells. A clinical example for the treatment of choroidal UM is belzupacap sarotalocan (AU-011) VDCs, which consist of a light-activated drug and VDCs that bind heparan sulfate proteoglycans on tumor cells, thereby inducing tumor cell necrosis following light activation and immune-mediated tumor cell killing [205,206,207]. Currently in phase 3 clinical trials, AU-011 (from Aura Biosciences) has shown an adequate safety profile following suprachoroidal administration [206]. Multiple clinical applications of AU-011 are proposed, such as tumor control [208] and better vision preservation in small tumors with a greater risk of vision loss with radiotherapy [209].

#### 8.1.3. Adoptive T Cell Therapy

Adoptive cell therapy (ACT) is a technique that extracts T cells from patients, which are then subsequently genetically modified and re-infused into patients to enhance the tumor-targeting characteristics [210]. Specifically for UM, this therapy revolves around the use of tumor-infiltrating lymphocytes (TILs) as well as genetically engineered T cells [192]. TILs are extracted from resected tumor tissues. The lymphocytes are primed for recognition of tumor-associated antigens, cultured in vitro in the presence of IL-2, and re-introduced into the patient [211]. They are then able to recognize cancer cells and subsequently destroy them. When TILs are removed from their tumor microenvironment, they have shown successful expansion along with UM-reactive T cells, which suggests T cell therapy can be utilized as an adjuvant treatment in UM with high risk of metastasis [191]. Recent trials have explored the efficacy of TIL therapy in metastatic UM. A phase II trial has shown that significant tumor regression can be induced in a subset of patients with TIL therapy, which suggests that the tumor microenvironment of UM can be manipulated to enhance T cell-mediated anti-tumor responses [192]. CAR T cell therapy involves chimeric antigen receptors (CAR), which natively transmit signals similar to those from activated T cell receptors [212]. They are subsequently genetically engineered to destroy cancer cells. A novel humanized mouse model demonstrated that CAR T cells can eliminate UM cells both in vitro and in vivo within xenografts grown in a NOD/SCID IL2 receptor gamma (NOG) knockout mouse strain transgenic for human IL2 [193]. It was also shown to be able to destroy melanoma cells resistant to ACT of autologous TILs, suggesting a viable option of therapy when UMs do not respond to standard therapy [193].

### 8.2. Gene Therapy

Gene therapy represents a cutting-edge approach aimed at correcting genetic defects that contribute to UM development. In the ensuing section, we will provide an overview of current gene therapies for the treatment of UM (Table 6).

#### 8.2.1. Suicide Gene Therapy

Suicide gene therapy entails introducing genes into cancer cells to increase their susceptibility to a pro-drug. It is specifically advantageous to selectively target tumor cells while avoiding damage to normal tissues [223].

The cytosine deaminase (CD) gene converts the antifungal drug 5-fluorocytosine (5-FC) into the cytotoxic chemotherapeutic agent 5-fluorouracil (5-FU) [224]. A study by Liu et al. showed that transfection of transcriptional suicide genes, such as CD, in the OCM-1 UM cell line significantly decreases cell proliferation and increases cell sensitivity to 5-FU [213]. Another study extracted primary UM cells and UM-associated fibroblasts from patients, cultivated them in vitro, followed by an infection with a retrovirus containing the suicide gene-fused yeast cytosine deaminase::uracil phospho-ribosyl transferase (*yCD::UPRT*) [215]. Small EVs were continuously produced with mRNA of the suicide gene in an expanded population of *yCD::UPRT*-UM cells with the integrated provirus. These sEVs were taken up by the tumor cells and were involved in the conversion of the 5-FC prodrug to the cytotoxic 5-FU. Another approach for suicide gene therapy is to target B7-H3, an immune checkpoint protein that is highly expressed in cancel cells such as UM [225,226]. Using a B7-H3 chimeric antigen receptor (CAR) with an inducible caspase-9 (iCas9) suicide gene, effective cell death of the UM cell line was shown, as well as a significant decrease in UM liver metastasis in murine models [214]. However, these results are limited to in vitro settings and must be replicated in animal preclinical studies.

#### 8.2.2. RNA Interference

RNA interference (RNAi) is a biological process in which RNA molecules inhibit gene expression, typically by causing the destruction of specific mRNA molecules [227]. Small interfering RNAs (siRNAs) and microRNAs (miRNAs) are used to target and silence genes that are critical for cancer growth and survival. In the context of UM, VEGF and Bcl-2—an antiapoptotic protein—are frequent targets [216]. Several RNAi-based therapies have shown potential in UM [228,229]. One study created hyaluronic acid (HA)-coated chitosan (Chi)/siRNA ternary complexes and targeted hypoxia-inducible factor 1α (HIF-1α), showing excellent cellular uptake and lysosome escape ability with low cytotoxicity, while inhibiting the invasive potential of UM via VEGF and HIF-1α down-regulation [216]. LncRNAs (i.e., long non-coding RNAs) interact with multiple proteins as well as DNA, often acting as scaffolds or guides [230]. Specific tumor suppressor lncRNAs, such as lncRNA, *Paupar*, or *Numb,* could be harnessed as therapeutic models for UM treatment. However, they are limited by both in vivo drug delivery and the lack of lncRNA-microRNA interactions described in the literature [217]. Another miRNA with demonstrated therapeutic potential in UM treatment is miR-181a, which was shown to be the solely downregulated miRNA among three studies of miRNA expression [218,219,220,221]. Despite widespread lncRNA–microRNA interactions in nature, very few have been described in UM. This is partially attributed to the rare nature of the disease as well as the novelty of the field of research. A database known as VECTOR (i.e., uVeal mElanoma Correlation NeTwORk) has been published to predict RNA interactions in UM [222]. Overall, RNA-based therapies show therapeutic potential but may present with more adverse effects due to their targeting of multiple genes. Another drawback is their low stability, endocytosis, and immunotoxicity, and they are rapidly degraded by nucleases [229].

## 9. Limits and Challenges in the Management of Uveal Melanoma

Managing UM presents numerous challenges. Firstly, the high metastatic potential of UM presents a challenge as it commonly metastasizes to the liver, often occurring despite successful local control of the primary tumor. New strategies for detecting and treating micrometastatic disease are critically needed. Furthermore, there are currently limited treatment options given the rarity of UM. Its unique features limit the availability of targeted treatments and the feasibility of large-scale clinical trials. The future of UM treatment lies in the integration of local control measures with systemic therapies, including immunotherapy and gene therapy, tailored based on genetic and molecular profiling of tumors. Early detection and management of metastatic disease are crucial for improving patient outcomes. Collaborative research and increased funding will be essential to address these challenges and develop more effective therapies for UM.

## 10. Conclusions

In conclusion, while current therapies provide effective local control, the high metastatic nature of UM and its resistance to conventional therapies highlight the urgent need for innovative treatment approaches. Further research needs to be performed to investigate the crucial role of CHCs in metastatic UM and niche formation. Furthermore, with the advances in liquid biopsies, continuous efforts need to be deployed for additional tumor marker identification for early disease diagnosis and disease progression. Immunotherapy and gene therapy represent promising frontiers but require further development and clinical validation to enhance their efficacy in the treatment of UM. The ongoing research and development in these areas hold significant potential to improve survival and quality of life for UM patients.

## Figures and Tables

**Table 1:** Summary of prognostic factors of uveal melanoma based on phenotypic presentation, molecular and genetic testing, and histopathological features ^a^.

Survival Rate	Low	Medium	High
Clinical	LBD > 16 mm Thickness > 3 mm Juxtapapillary Ciliochoroidal Extrascleral extension	LBD 6–16 mm Thickness 1–3 mm	LBD < 6 mm Thickness < 1 mm Iris
Molecular ^b^	Loss of 6p, 6q, and 8p; 8q gain	N/A	6p gain
Histopathological	Epithelioid-cell type, infiltrating lymphocytes, increased mitotic activity	Mixed-cell type	Spindle-cell type
Genetic	Class 2, *PRAME* mutation	Class 1B	Class 1A

^a^ Abbreviations: LBD; large basal diameter, mm; millimetre, *PRAME*; PReferentially expressed Antigen in Melanoma, N/A; not applicable. ^b^ Molecular associations occurring in chromosome 3 monosomy tumors.

**Table 2:** Summary of clinical and multimodal imaging characteristics of indeterminate nevi with risk of malignant transformation.

Variable	Clinical Feature	Growth Correlation
T	Thickness > 2 mm	Positive
F	Fluid (subretinal)	Positive
S	Symptoms	Positive
O	Orange pigment	Positive
M	Margin > 3 mm to disc	Positive
UH	Ultrasound hollow	Positive
H	Halo absent	Positive
D	Drusen absent	Negative

**Table 3:** Summary of uveal melanoma markers used in liquid biopsy.

Marker Type	Findings	References
Circulating tumor cells (CTCs)	Used for disease prognostication Increased concentrations are associated with worse prognosis Used for discrimination between uveal melanoma and nevi	[112,113,114,115,116]
Circulating tumor DNA (ctDNA)	Monitoring predicts disease response and metastatic uveal melanoma progression Positively correlates with metastatic hepatic disease Association with CTCs and progression-free survival	[117,118]
Circulating micro-RNA (miRNA)	Increased plasma miRNA-618 and decreased vitreous miRNA Differential regulation in metastatic versus non-metastatic disease	[119,120]
Melanoma-specific gp100	Upregulated	[121,122]
Cathepsin	Upregulated	[121,122]
Heat shock protein 27	Differentiate between metastatic and non-metastatic UM post treatment	[123]
Osteopontin	Differentiate between metastatic and non-metastatic UM post treatment	[123]
S-100 protein	Upregulated in both the vitreous and aqueous humor	[124]

**Table 5:** Summary of most recent advances in immunotherapy for the management of uveal melanoma.

Agent	Study Type	Main Findings	References
**Checkpoint Inhibitors**			
Ipilimumab	N/A	At 3 mg/kg, overall response rates (ORR) were of 0 to 4.8%. Higher doses (10 mg/kg) provided longer median overall survival rates, but similar overall response rates compared to lower doses.	[170,171,172,173,174,175]
Pembrolizumab Nivolumab Ipilimumab	Retrospective cohort study Clinical trials (NCT02626962.P1)	Less effective in UM than cutaneous melanoma due to lower mutational burden. Checkpoint inhibitors show limited effectiveness in UM due to a lower number of neoantigens. Combined nivolumab and ipilimumab showed a median OS of 12.7 months. Systemic therapies showed a median OS of 9.3 months.	[176,177,178,179,180,181,182,183]
Tebentafusp	Previously untreated HLA-A*0201-positive patients with metastatic uveal melanoma	Demonstrated overall survival benefit in metastatic UM but limited to patients who are HLA-A*0201 positive.	[10]
Dual checkpoint inhibitors	Meta-analysis	Dual checkpoint inhibitors are more effective than single agents for metastatic UM.	[184]
**Oncolytic viruses**			
T-VEC	In vitro UM cell lines Clinical trials (NCT02509507)	Showed potential with local control and durable systemic response. Alters tumor microenvironment to enhance immune attack.	[185,186]
ECHO-7 Coxsackieviruses HF-10	In vitro UM cell lines	These viruses are being explored for efficacy in UM, with promising results in initial studies.	[186,187]
HSV-EGFP VSV-IFNβ-TYRP1	In vitro UM cell lines Clinical trials	Demonstrated effectiveness in vitro and in vivo. VSV-IFNβ-TYRP1 is safe in patients with metastatic UM. Combination with checkpoint inhibitors enhances immune response in patients with metastatic UM (coxsackie (CAVATAK) combined with Ipilimumab).	[188,189,190]
**Adoptive T Cell therapy**			
TIL therapy	TILs from primary UM NOD/SCID IL2 receptor gamma (NOG) knockout mouse strain	Induced significant tumor regression in a subset of patients, suggesting manipulation of tumor microenvironment can enhance anti-tumor responses. TILs show potential as an adjuvant treatment for UM with high metastatic risk. CAR-T cells effective in vitro and in vivo against UM cells and resistant tumors in specific mouse models.	[191,192,193]

**Table 6:** Overview of gene therapy methods for the treatment of uveal melanoma.

Gene Therapy	Model Used	Main Findings	References
Cytosine deaminase (CD) gene therapy	Murine models with genetically engineered CD OCM-1 cells	Introduction of the CD gene makes tumors sensitive to 5-FU.	[213]
B7-H3 CAR T cells with iCas9	Human UM tissue samples and cell lines	Created B7-H3 CAR T cells with an inducible caspase-9 suicide gene demonstrated a durable anti-tumor response.	[214]
yCD::UPRT gene therapy	In vitro primary UM cells and associated fibroblasts	Transduction with yCD::UPRT gene leads to production of sEVs carrying the suicide gene, showing potential for targeting UM cells. Needs further validation in animal models.	[215]
RNA Interference (RNAi)			
siRNAs and miRNAs targeting VEGF and Bcl-2	Human UM cell line MP-38 (ATCC CRL-3296)	RNA molecules, such as siRNAs and miRNAs, are utilized to target and silence genes critical for cancer growth, particularly VEGF and Bcl-2 in the context of UM.	[216]
HA-coated chitosan/siRNA complexes targeting HIF-1α	Human UM cell line MP-38 (ATCC CRL-3296)	Demonstrated excellent cellular uptake and lysosome escape, with low cytotoxicity, effectively inhibiting the invasive potential of UM by down-regulating VEGF and HIF-1α.	[216]
LncRNAs as therapeutic agents (PAUPAR, NUMB)	N/A	They have therapeutic potential in UM but face in vivo drug delivery challenges and lack of described interactions.	[217]
miR-181a	Clinically defined UM samples	Identified as solely downregulated miRNA among three studies, showing significant potential as a therapeutic target in UM.	[218,219,220,221]
VECTOR database	VECTOR (uVeal mElanoma Correlation NeTwORk) database	Published to predict RNA interactions in UM, addressing the rarity of described lncRNA–microRNA interactions and aiding in the study of RNA based therapies.	[222]
siRNAs and miRNAs targeting VEGF and Bcl-2	Human UM cell line MP-38 (ATCC CRL-3296)	RNA molecules, such as siRNAs and miRNAs, are utilized to target and silence genes critical for cancer growth, particularly VEGF and Bcl-2 in the context of uveal melanoma.	[216]
